# A Study on Corrosion Inhibitor for Mild Steel in Ethanol Fuel Blend

**DOI:** 10.3390/ma11010059

**Published:** 2017-12-31

**Authors:** Nguyen Si Hoai Vu, Pham Van Hien, Tran Van Man, Vu Thi Hanh Thu, Mai Dinh Tri, Nguyen Dang Nam

**Affiliations:** 1 Department of Physics & Engineering Physics, University of Science, VNU-HCM, 227 Nguyen Van Cu Street, District 5, Ho Chi Minh City 700000, Vietnam; ngsh.vu@gmail.com; 2 Faculty of Chemical Engineering, Bach Khoa University, VNU-HCM, 268 Ly Thuong Kiet Street, District 10, Ho Chi Minh City 700000, Vietnam; phamvanhien240991@gmail.com; 3 Department of Physical Chemistry, University of Science, VNU-HCM, 227 Nguyen Van Cu Street, District 5, Ho Chi Minh City 700000, Vietnam; tvman@hcmus.edu.vn; 4 Institute of Chemical Technology, Vietnam Academy of Science and Technology, 1 Mac Dinh Chi Street, District 1, Ho Chi Minh City 700000, Vietnam; maidinhtri@gmail.com; 5 External Relations and Science & Technology Department, PetroVietnam University, Cach Mang Thang Tam Street, Long Toan Ward, Ba Ria City 790000, Vietnam

**Keywords:** corrosion inhibitor, ethanol fuel blend, aganonerion polymorphum, leaf extraction, electrochemistry

## Abstract

The main aim of this study is to investigate Aganonerion polymorphum leaf-ethyl acetate extract (APL-EAE) and its inhibiting effect for steel in ethanol fuel blend. The immersion test, electrochemical and surface analysis techniques were successfully carried out in this research. Scanning electron microscope images indicated that the ethanol fuel blend induced pitting corrosion of steel. Remarkably, the surface of the sample containing 1000 ppm APL-EAE is smoother than the others submerged in different conditions. The electrochemical impedance spectroscopy result shows that APL-EAE has formed a good protective layer, preventing corrosive factors from hitting the steel surface. The potentiodynamic polarization data argue that the corrosion inhibition efficiency was strengthened with the increase of APL-EAE concentration. The Fourier transform infrared spectroscopy and X-ray photoelectron spectroscopy demonstrated less intensity of Fe peaks, higher intensity of C_1s_ peak and the appearance of organic peaks (N_1s_, P_2p_, O_1s_) from specimens with and without APL-EAE addition. Therefore, the results suggest the formation of the protective film on steel surface and affirm that APL-EAE has served as an effective corrosion inhibitor for steel in ethanol fuel blend.

## 1. Introduction

Recently, global warming and drastic changes in climatic conditions have been attracting more attention of governments. Scientists manifested that these phenomena have mainly brought the rise of greenhouse gas (CO_2_, CH_4_, and N_2_O) emission due to human activities in which above half of carbon dioxide in the atmosphere originates from the burning of fossil fuels over the past 20 years [1,2]. In addition to this, the exploitation and transportation of fossil fuels also generate environmental pollution issues that have significant impacts on ecosystems. Indeed, it has become imperative to reduce utilization of fossil fuels by encouraging alternative sources such as biofuels. However, because of limitations of current technology, the complete replacement of fossil fuels with biofuels is infeasible. To address this issue, the best possible solution is introducing ethanol fuel blend, as the mixture of bio-ethanol and traditional gasoline. Many blend levels of anhydrous bio-ethanol in ethanol fuel blend was labeled as E number; for example, well-known E10 fuel means there was 10% of bio-ethanol and 90% of gasoline in a unit of volume. Due to the significant advantages, many countries have allowed commercial ethanol fuel blend because of its high octane number, better thermal efficiency, complete combustion, less greenhouse gas emission, and more environmentally friendly than fossil fuels [3,4]. In contrast, there were some disadvantages, such as culpable, aggressive corrosion of petroleum pipeline systems and storage tanks, mainly being made of steel. Firstly, bio-ethanol is hygroscopic; this increases water content in the fuel, engendering electrochemical corrosion. Secondly, due to its origin and manufacturing process, bio-ethanol has a lot of corrosive contaminants such as ions (chloride and sulfate ions) or organic acids (formic and acetic acids), resulting in pitting corrosion. Third, bacteria in the blend can produce some acids, salts or ammonia, acting as corrosive agents, which are responsible for microbial influenced corrosion. There have been many reports about corrosion of steel in petroleum pipelines and storage tanks being induced by ethanol fuel blend [5,6,7,8].

Steel has been widely used in many industries, attributed to its relatively low cost and high mechanical strength, but regrettably, it is readily prone to corrosion [9]. Since steel plays a crucial role in petroleum industry, companies have prioritized finding ways of protecting steel rather than replacing it by other materials. Some of the ways consist of alloys, coatings, special treatments or inhibitors [10,11,12,13,14,15,16,17], in which the inhibitors have been demonstrated possessing the most suitable advantages. Most of today’s commercial corrosion inhibitors are amine-based as well as its derivatives. Unfortunately, these substances have high toxicity; for instance, low molecular weight aliphatic amines are easily ingested through the skin, and aromatic amines are carcinogenic [18,19]. Therefore, it is necessary to seek for a new generation of inhibitors for ethanol fuel blend, focusing on efficiency, environment and human health friendliness, heading to alternate amines. Lots of recent reports show a research trend in the use of organic inhibitors related to natural extraction, especially leaf extracts, as green inhibitors for steel in bio-diesel, seawater and acidic medium [20,21,22]. Nevertheless, they rarely have comparable reports on green inhibitors in ethanol fuel blends as well as almost no reports on the usage of aganonerion polymorphum extraction as a corrosive inhibitor; thus it became the motivation of the current research.

The mechanism of such inhibitors, in short, is the constitution of a protective layer onto steel surfaces to separate the corrosive medium and the metal substrate, which was revealed by previous articles [23,24]. Investigation of this layer requires modern techniques such as electrochemical analysis to figure out the formation process along with the attributes of the protective film. However, due to very high impedance of these types of fuels, electrochemical measurements have hardly been applied directly to the solution without adding conductive salts. Therefore, Liu Cao et al. added LiCl to ethanol fuel blend to increase the conductivity of the mixture [25], or the mensuration was modified according to Hassan Jafari et al. [9]. The intervention of them may alter the characteristics of the original solution, and consequently, the outcomes should be carefully dissected.

In this study, we focus on clarifying the inhibiting efficacy and the protective mechanisms of Aganonerion polymorphum leaf-ethyl acetate extract (APL-EAE) eco-friendly inhibitor on steel in ethanol fuel blend. All of the experimental processes and data analyses in this work followed American society for testing and materials (ASTM) standards. Due to a low corrosion rate of the blend (on steel), simulated ethanol fuel blend was engaged with an accelerated laboratory test method (ASTM D7577) [26]. State-of-the-art analysis techniques are utilized as described below. The electrochemical analysis without any modification of the original environment displays the existence of deposited films. Support of surface analysis techniques such as Scanning electron microscope (SEM), Attenuated total reflectance Fourier-transform infrared spectroscopy (ATR FT-IR) and X-ray photoelectron spectroscopy (XPS) certifies the film together with its contents.

## 2. Experimental Details

### 2.1. Materials

Aganonerion polymorphum leaves (APLs) derived from the southeast Vietnam rain forest were washed to remove mud and soil, dried in an oven at 60 °C, and carefully triturated to powder. Then, 100 g of APLs powder were added to Soxhlet extractor containing ethyl acetate (EA) (minimum purity 99.5%, Merck Pte Ltd, Singapore, Singapore), then being extracted at 75 °C for 24 h. The role of EA and extracted temperature is thought to construe mid-polarized substances and elimination of heat-unstable substances, and help better dissolution and longevity of the extraction. The extract (APL-EAE) then was filtered through filter paper, concentrated and removed the solvent in the IKA RV-10 Rotary Evaporator system (RV 10 digital V, Vietnam Academy of Science and Technology, Ho Chi Minh City, Vietnam), and finally stored in the dry cabinet at room temperature.

Steel was CNCed to several 10 mm × 10 mm × 3 mm specimens. Then, these specimens had been treated under ASTM G1 [27] to remove corrosion products after previous tests and to evaluate the corrosion damage of the metal surface, then were wired and moulded by Epoxy resin (Agar Scientific Ltd., London, UK) under ASTM G31 [28]. Final samples have 10 mm × 10 mm of surface area, and were ground by 2000 grit silicon carbide paper before using.

### 2.2. Immersion Test

The immersion test was taken under ASTM D7577 rating No. 5 [26], and the simulated ethanol fuel blend contents are described in Table 1. In this paper, commercial unleaded gasoline RON92 was used instead of fuel C, and all chemicals are reagent grade, purchased from Merck and used directly without any purification. Testing solutions contain the inhibitor (APL-EAE) with different concentrations 0, 100, 500, 1000 and 1500 ppm. The mixtures had been seriated and magnetically stirred at 900 rpm at 38 °C for an hour in closed beakers, and then stabilized for 15 min before the immersion process was employed. Finally, the samples were inserted into the beakers with their metal faces downwards. The immersion test lasted 24 h in a closed cabinet. The electrochemical tests followed right after finishing immersion test without any touch to the previous solution.

### 2.3. Electrochemical Test

Electrochemical impedance spectroscopy (EIS) and potentiodynamic polarization (PD) measurements were manipulated by the VSP Potentiostat (Bio-Logic Science Instruments, Seyssinet-Pariset, France), the setting parameters follow ASTM G5 [29] with two platinum meshes as counter electrode, and the silver/silver chloride as reference electrode. For EIS measurement, the peak-to-peak amplitude of the sinusoidal perturbation was 10 mV, and the frequency varied from 100 kHz to 10 mHz. The PD measurement went after the EIS analysis, and the scan range ran from −0.250 V vs. open circuit voltage (E_OCP_) to 800 mV vs. V_Ag/AgCl_ with scan rate is 0.166 mV/s. EIS, PD data and fitting parameters were handled and calculated by default functions of EC-Lab commercial software (version 10.36, Bio-Logic Science Instruments, Seyssinet-Pariset, France) integrated with the VSP system.

### 2.4. Surface Analysis

The steel components were determined by optical emission spectroscopy (OES) as given in Table 2. Scanning electron microscope (SEM) images were recognized by Hitachi S-4800 Field Emission Scanning Electron Microscope (Institute for Nanotechnology, Ho Chi Minh City, Vietnam). After 24 h of immersion test at different APL-EAE concentrations, the samples were dried, and the FT-IR spectroscopy of the samples was taken by attenuated total reflectance (ATR)-extended part of the IR Tracer 100 Fourier Transform Infrared Spectrophotometer, Shimadzu system (Institute for Nanotechnology, Ho Chi Minh City, Vietnam). XPS data of the samples without and with 1000 ppm APL-EAE after 24 h of immersion test were also examined by AES-XPS ESCA-2000 system (Sungkyunkwan University, Seoul, Korea).

## 3. Results and Discussion

### 3.1. Surface Analysis

**Figure 1:**
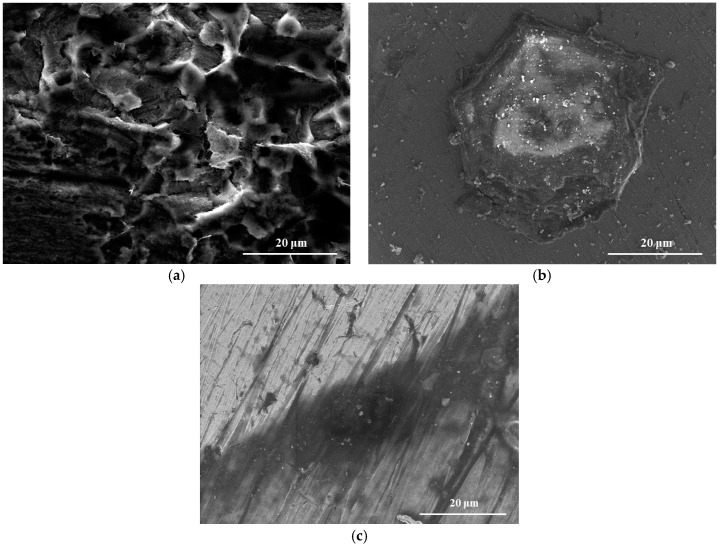
Scanning electron microscope (SEM) images of steel surface after 24 h of immersion test in a simulated environment (**a**) without inhibitor; (**b**) with 100 ppm of APL-EAE; and (**c**) with 1000 ppm of APL-EAE.

To investigate the change of surface morphology in the test, SEM examination was applied, and results are given in Figure 1.

Figure 1a exhibits the rough surface of the sample without protection of APL-EAE; some pitting holes are surrounded by clusters of substances which may be related to rusting product. In contrast, samples with the protection of APL-EAE (Figure 1b,c) show less pitting holes and the advent of a protective layer upon steel surface. With 1000 ppm of APL-EAE concentration, the grinding strains are clearly visible, demonstrating a good protecting effect, as shown in Figure 1c. It was necessary to analyze the composition of that layer; therefore, ATR FT-IR and XPS analysis of the samples were applied and details are discussed below.

**Figure 2:**
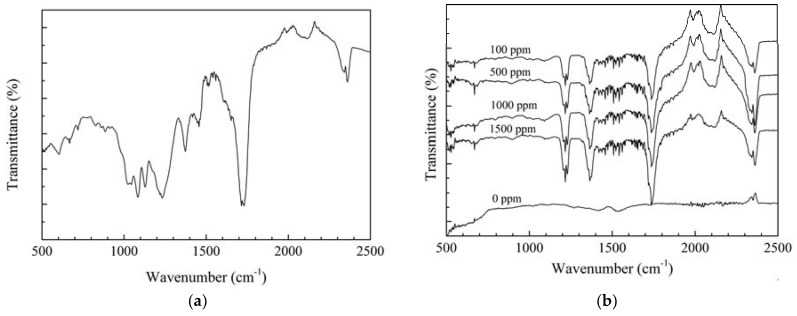
ATR FT-IR results of (**a**) as-prepared Aganonerion polymorphum leaf-ethyl acetate extract (APL-EAE) glue; and (**b**) steel surfaces after 24 h immersion in simulated ethanol fuel blend solutions containing different APL-EAE concentrations.

The ATR FT-IR method was utilized to evaluate the chemical properties of the protective film deposited on the steel surface where the main components are organic compounds. Figure 2a shows the ATR FT-IR results of the as-prepared APL-EAE glue. The peaks at 2362 cm^−1^ and 2343 cm^−1^, 1715 cm^−1^, 1520 cm^−1^ and 1233 cm^−1^ are assigned to the O–C=O groups, the C=O bond, and the aromatic ring, respectively. Besides, the peaks at 1375 cm^−1^, 1084 cm^−1^ and 1028 cm^−1^ are related to, respectively, the C−H bending mode in methyl group, the asymmetric stretching mode of C–O−C, and the stretching mode of C–N. The bands below 1000 cm^−1^ indicate to the aliphatic and aromatic C−H groups [30,31,32]. Figure 2b shows the ATR FT-IR spectra of the samples immersed in the ethanol fuel blend environment containing various inhibitor concentrations. It expresses the existence of the absorption bands of the O–C=O group (at 2362 cm^−1^ and 2338 cm^−1^), the C=O (at 1737 cm^−1^), the C−H (at 1366 cm^−1^), the aromatic ring (at about 1510 cm^−1^ and 1219 cm^−1^), the aliphatic and aromatic C–H group (below 1000 cm^−1^). These results also recorded no peaks of rusting products or other organic compounds in the samples without inhibitor while the intensity of these peaks increases with the increase of APL-EAE concentration. In conclusion, the ATR FR-IR spectra confirm the formation of an organic layer on the samples immersed in APL-EAE.

**Figure 3:**
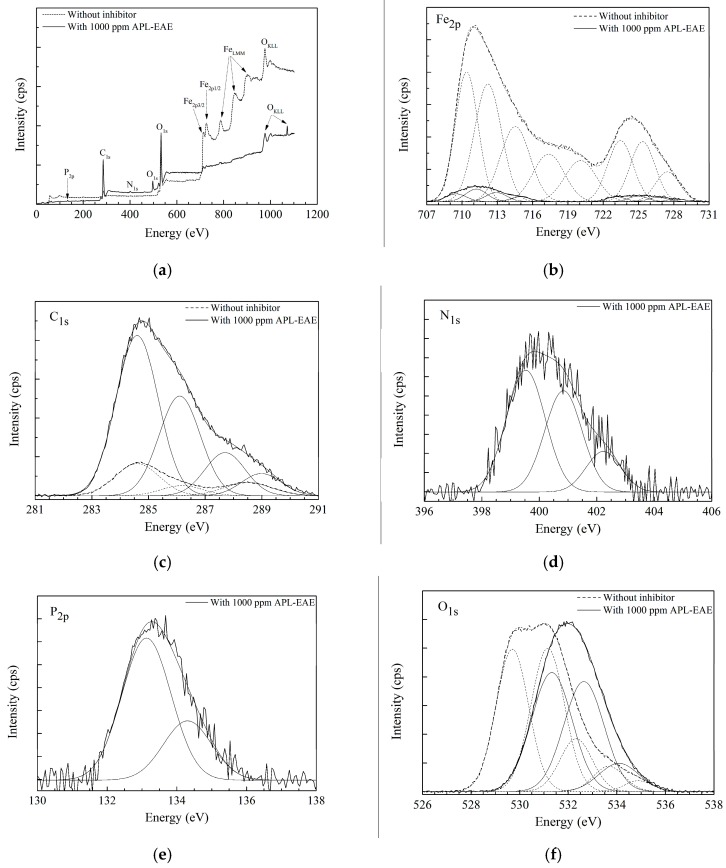
(**a**) Survey scan spectrum of XPS measurement, showing the growth of the protective film on the steel surface peak. Narrow scan spectra of (**b**) Fe; (**c**) C; (**d**) N; (**e**) P; and (**f**) O coverage of steel surface determined by XPS.

To investigate the elemental constitution and the chemical state of the deposited layer, as well as to detect the attendance of iron oxides on the sample surface that cannot be detected in ATR FT-IR spectra due to its high binding energies, the XPS analysis was employed. Figure 3 displays XPS results of samples after 24 h immersion test of steel in simulated ethanol fuel blend solution without and with 1000 ppm APL-EAE. The wide-range XPS plot of the samples in Figure 3a exhibits high iron peaks from 700 eV to 950 eV of the sample without inhibitor, whereas there are no similar peaks on the sample with inhibitor. Besides, Figure 3a also shows significantly a higher peak of C_1s_ near 300 eV as well as the appearance of three new peaks of O_1s_, N_1s_ and P_2p_ on, respectively, at 500 eV, 400 eV and near 150 eV in inhibited system. The Fe, C_1s_, N_1s_, P_2p_ and O peaks in XPS data indicate the existence of iron, carbon, nitrogen, phosphorus and oxygen elements in chemical bonding on the sample surfaces. The difference from the XPS plot proves that APL-EAE indeed had a positive influence on the surface reactions of the sample. The narrow-scan plot of Fe in Figure 3b reveals two strong peaks of a mixture of ferric and ferrous oxides (Fe_3_O_4_, Fe_2_O_3_, and FeO) at 711.2 eV and hydrated ferric oxide (FeOOH) at 724.4 eV in the uninhibited system. However, there has been relatively lower intensity of these Fe peaks in the inhibited system. That means APL-EAE successfully blocked the steel surface and less rusting products were created. The binding energy of C_1s_ indicates the presence of signals from carbon contamination (C, C−C, C=C) at 284.6 eV, C–O at 286.1 eV, C=O at 287.7 eV, and O−C=O at 289.0 eV, as shown in Figure 3c. These peaks, apparently, are organic substances that came from the ethanol fuel blend environment and APL-EAE [33,34].

The significantly higher peak of C_1s_ as well as two new N_1s_ and P_2p_ peaks (Figure 3d,e, respectively) in the inhibited system may be contributed by APL-EAE. Besides, the O_1s_ peak in Figure 3f also demonstrates the signals, indicating the products of oxide, hydroxyl, water on the steel surface, respectively, at 529.7 eV, 531.1 eV and 532.3 eV, 533.7 eV and 533.9 eV in the uninhibited system; and at 531.3 eV, 532.6 eV, 534.2 eV in inhibited system. The shifted peaks suggest the change in chemical bonding of oxygen, from iron oxide states to organic bonding states in APL-EAE. The XPS data, in conclusion, confirm that the film formation via the adsorption of APL-EAE on the sample surface by the combination of steel produced the complex product of APL-EAE and iron, covering the sample surface and preventing the attack of corrosion agents and the dissolution of steel.

### 3.2. Electrochemical Analysis

EIS data give lots of information on corrosion and inhibiting properties of a system consisting of the issue as to whether the protective film on the metal surface has inhibiting properties or not, the properties of the deposited layer, the properties of the interaction between the deposited layer and the metal substrate. In this study, APL-EAE from the solutions continuously combined with iron ions which were released from corrosion reactions, then being deposited onto the substrate and blocked this site. The major constituent of the inhibiting product formed a protective layer while the others sealed the pores containing corrosion solution at the interfaces of the deposited layer and the metal substrate, which can be analyzed by EIS measurement.

**Figure 4:**
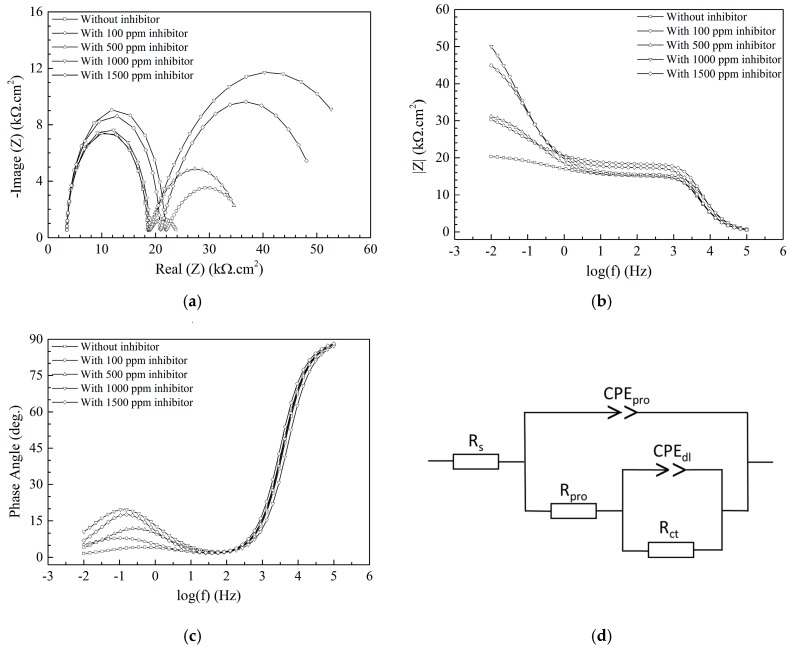
(**a**) Nyquist; Bode plots of (**b**) |Z| versus frequency; and (**c**) phase angle versus frequency of steels after 24-hour immersed in simulated ethanol fuel blend solutions containing different APL-EAE concentrations; and (**d**) equivalent circuit for fitting the impedance data.

Figure 4a shows the Nyquist plot of samples with different APL-EAE concentrations (0, 100, 500, 1000, and 1500 ppm); there are two semicircles appearing in the Nyquist plot. In addition, the two Bode plots, respectively, total impedance vs. frequencies (Figure 4b) and phase angle vs. frequencies (Figure 4c), show a phase that has low impedance, high phase angle in the range of 10^3^–10^5^ Hz; as well as a second phase that has high impedance, low phase angle in the range of 10^−2^–10^1^ Hz. It can be concluded that there are two phases existing in the deposited layer, representing the layer of the inhibitor and the interaction between that layer and the substrate surface. Consequently, the circuit model as shown in Figure 4d is proposed to fit the Nyquist plot, while R_s_ is the solution resistance, CPE_pro_ is the capacitance of the deposited layer (rust layer in uninhibited system and inhibitor layer in inhibited one), R_pro_ is the resistance of the pores of the deposited coating, CPE_dl_ and R_ct_ are the capacitance of the double layer and the resistance of the charge transfer between the deposited layer and the metal substrate, respectively. Nyquist plot fitting data for APL-EAE-containing-samples were extrapolated using the equivalent circuit as described in Figure 4d. All results have extreme low relative error (χ^2^ below 0.2%), indicating good fitting quality. The first semicircle in Figure 4a indicates a characteristic of the protective layer of the uninhibited system and inhibited system containing various concentrations of APL-EAE. R_pro_ values of the first semicircle dramatically increase from 0 ppm to 100 ppm APL-EAE (from 14.95 kΩ to 18.14 kΩ, specifically) due to the significant mixture of rusting product in combination with the discontinuous inhibiting layer. However, this mixture was unstable because of poor properties of the contact layer, which is recorded in the second semicircle. When the APL-EAE concentrations varied from 500 ppm to 1500 ppm, rusting products were significantly inhibited, thus, the deposited film was mostly covered by the layer of the inhibitor (APL-EAE), which was more stable than the previous mixture (R_pro_ ranges from 21.74 kΩ to 44.18 kΩ). R_pro_ values are significantly increased in inhibited system containing inhibitor concentrations ranging from 500 ppm to 1500 ppm (18.14, 44.18, 26.51 kΩ, respectively), demonstrating the formation procedure of a stable and continuous layer that prevented the dissolution of steel by adherently depositing and canceling out the conduction paths inside the inhibitor layer. In addition, the second semicircle in Figure 4a maps to the presence of the interaction between the deposited layer and the metal substrate, showing the growth of the impedance values with the increasing of corrosion inhibitor concentration (R_ct_ raises from 5.86 kΩ to 18.14 kΩ), but there is the decrease in case of 1500 ppm APL-EAE (R_ct_ drops from 18.14 kΩ to 17.48 kΩ).

It can be concluded that when the higher inhibitor concentration was added, the better connection between the inhibitor layer and the metal substrate was archived, and the best protection effectiveness is recorded at 1000 ppm APL-EAE. However, at higher inhibitor concentration (1500 ppm), the disorderly formation of the adsorption components promoting the organization of some minor pores decreased the impedance value of the sample. As seen in Table 3, all CPE_pro_ values are 3 times of magnitude higher than CPE_dl_ values, as well as α_pro_ values range from 0.469 to 0.743 (relatively far from 1); this is evidence proving that the protective layer has a porous structure with insulating phases intermingled with conducting paths. In contrast, α_dl_ values are very close to 1 which means good interaction between the inhibition layer and steel substrate.

**Figure 5:**
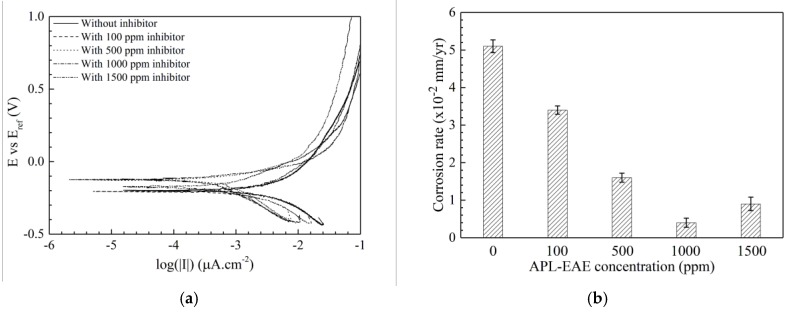
(**a**) Potentiodynamic polarization curves; and (**b**) effect of APL-EAE concentrations on the corrosion rate of steel after 24-hour elapsed for 1 cm^2^ immersed in simulated ethanol fuel blend solutions with initial APL-EAE concentrations from 0 ppm to 1500 ppm.

The corrosion behaviors of the samples then were evaluated by PD measurement. From the measured PD data, this result can infer some parameters such as polarization resistance, corrosion rate, and inhibition efficiency. Figure 5 displays the potentiodynamic curves of steel in ethanol fuel blend medium in the sample without inhibitor (uninhibited system) and with different APL-EAE concentration (inhibited systems). Calculated parameters from Tafel extrapolation of PD measurement are described in Table 4. *i_corr_* value significantly decreases at higher inhibited system (from 2.17 µA/cm^2^ of the sample without inhibitor to 0.17 µA/cm^2^ of the sample with 1000 ppm APL-EAE), associated with the mitigation of corrosion reactions by the action of APL-EAE inhibitor. Also, the *E_corr_* becomes nobler, and current density of cathode curves decreases with increasing corrosion inhibitor addition, indicating that APL-EAE primarily acts as a mixed-type leaning towards the anodic inhibition process. The polarization resistance, as well as the corrosion rate, were calculated following the corrosion current density following Equations (1) [35] and (2) [36], and the results are given in Table 3 and Figure 5b, respectively.

(1) $$R_{p}=\frac{1}{i_{corr}}\times \frac{\beta_{a}\beta_{c}}{2.303\left(\beta_{a}+\beta_{c}\right)}$$

where *R_p_* is the polarization resistance, *i_corr_* is the corrosion current density, *β_a_* and *β_c_* are the anodic and cathodic Tafel constant, respectively.

(2) $$C.R.=\frac{i_{corr}.k.EW}{d.A}$$

where *C.R.* is the corrosion rate in mm/yr, *i_corr_* is the corrosion current density (µA/cm^2^), k is a constant that specifies the units for the corrosion rate (*k* = 3.27 × 10^−3^ in mm), *EW* and *d* is the equivalent weight and the density of steel, respectively, and *A* is exposed area. The formula, *k* value and method to examine *EW* of steel in (2) followed ASTM G102 [36].

The polarization resistance in Table 4 expresses the increasing trend with the accession of the inhibitor, in combination with the increase of *E_corr_* and the decrease of *i_corr_* as mentioned above, promoting the improved inhibition performance at higher APL-EAE concentrations. In addition, two Tafel constants, *β_a_* and *β_c_*, reduce with the increase of inhibitor concentration, proving the formation of a layer onto the samples. Both anodic and cathodic branches of Tafel plot have the same change rule, which means APL-EAE acts as a mixed inhibitor. The inhibited system of 1000 ppm APL-EAE shows the lowest corrosion current density, highest corrosion potential, most depressed Tafel *β_a_* and *β_c_* constants, and highest polarization resistance that verify superior inhibition concentration than the others in this study. The values are, respectively, 0.17 µA/cm^2^, −125 mV_Ag/AgCl_, 80 and 211 mV/decade, 148.19 kΩ.cm^2^, and 0.004 mm/yr.

The results of this work sketched out a schematic formation of a coherent, stable passive inhibition layer of APL-EAE onto the steel surface, by electrostatic interaction between the organic molecules of the inhibitor and the charged iron ions released from the substrate after the corrosive reaction had occurred. The progressively better protective film was fulfilled by the more inhibitor concentration added, and the finest film structure is achieved at 1000 ppm APL-EAE. At this concentration, the inhibitor almost blocked the metal surface, hence significantly reducing rusting products. Besides, the most positive corrosion inhibition parameters, such as polarization resistance and corrosion rate, are also recorded at 1000 ppm APL-EAE concentration.

## 4. Conclusions

This work showed the ability to use APL-EAE as an effective, green mixed corrosion inhibitor for steel in a simulated ethanol fuel blend environment. APL-EAE formed an organic protective layer to facilitate the protection of the steel surface as apparent in ATR FT-IR images, with absorbance peak intensities increasing with the increasing of inhibitor concentration. XPS data show less Fe peak intensity, a significantly higher of C_1s_ peak, as well as the appearance of new organic peaks O_1s_, N_1s_, and P_2p_ on the sample in inhibited system containing 1000 ppm APL-EAE. The EIS data confirm the protective layer at 1000 ppm of APL-EAE is a stable film, having high impedance and phase angle, suggesting good adhesion to the steel substrate as shown in the Nyquist plot and the two Bode plots. Besides, Tafel fitting from PD measurement noticed the inhibited system of 1000 ppm APL-EAE has the lowest corrosion current density, highest corrosion potential, most inferior Tafel *β_a_* and *β_c_* constants, highest polarization resistance, and lowest corrosion rate, which are the best values of this study and have a good agreement with all above analysis results.

## Figures and Tables

**Table 1:** The simulated ethanol fuel blend contents.

Chemical	Origin	Minimum Purity (%)	Proportion
Ethanol	Merck	99.8	75.6% *v*/*v*
Methanol	Merck	99.8	4.2% *v*/*v*
Iso-Propanol	Merck	99.5	4.2% *v*/*v*
RON92	Commercial, unleaded	-	15% *v*/*v*
Deionized water	ELGA Purelab Ultra	ASTM D1193	1% *v*/*v*
Sodium chloride	Merck	99.5	15 ppm
Formic acid	Merck	95.0	10 ppm
Acetic acid	Merck	99.7	20 ppm

**Table 2:** Steel components determined by optical emission spectroscopy.

Chemical Elements (%)														
C	Mn	Si	S	P	Ni	Cr	Mo	Cu	V	Nb	Ti	Al	B	Fe
0.16	0.73	0.21	0.01	0.02	<0.01	0.03	<0.01	<0.01	0.01	<0.01	<0.01	<0.005	<0.005	Bal.

**Table 3:** Parameters obtained from the Nyquist plot fitting of steel after 24-hour immersing in simulated ethanol fuel blend solutions containing different APL-EAE concentrations following the equivalent circuit in Figure 4d.

Concentration (ppm)	R_s_ (kΩ.cm^2^)	CPE_pro_ (µF/cm^2^)	Α (0~1)	R_pro_ (kΩ.cm^2^)	CPE_dl_ (nF/cm^2^)	Α (0~1)	R_ct_ (kΩ.cm^2^)	χ^2^ (%)
0	3.52	140.0	0.442	14.95	21.4	0.899	5.86	0.136
100	3.56	106.0	0.569	18.14	15.2	0.939	15.14	0.161
500	3.55	68.1	0.604	21.74	4.9	0.982	15.80	0.182
1000	3.58	50.1	0.743	44.18	3.0	0.998	18.14	0.116
1500	3.68	58.4	0.617	26.51	3.9	0.994	17.48	0.170

**Table 4:** Corrosion parameters obtained from the potentiodynamic polarization curves of steel after 24-hour immersed in simulated ethanol fuel blend solutions containing different APL-EAE concentrations.

Concentratio*n* (ppm)	*E_corr_* (mV_Ag/AgCl_)	*i_corr_* (µA/cm^2^)	*β_a_* (mV/decade)	*β_c_* (mV/decade)	*R_p_* (kΩ.cm^2^)
0	−198	2.17	377	295	33
100	−206	1.46	296	304	45
500	−127	0.69	133	275	56
1000	−125	0.17	80	211	148
1500	−173	0.39	172	255	114
